# Neuropsychiatric Symptom Profile in Alzheimer’s Disease and Their Effects on Functional Decline

**DOI:** 10.1002/alz.086695

**Published:** 2025-01-03

**Authors:** Carolyn W. Zhu, Lon S. S. Schneider, Amy Aloysi, Gregory Elder, Hillel Grossman, Judith A. Neugroschl, Corbett Schimming, Laili Soleimani, Mary Sano

**Affiliations:** ^1^ James J. Peters VA Medical Center, Bronx, NY USA; ^2^ Icahn School of Medicine at Mount Sinai, New York, NY USA; ^3^ Alzheimer’s Disease Research Center, Keck School of Medicine, University of Southern California, Los Angeles, CA USA; ^4^ Department of Psychiatry and Behavioral Sciences, Keck School of Medicine, University of Southern California, Los Angeles, CA USA; ^5^ Keck School of Medicine of USC, Los Angeles, CA USA; ^6^ James J Peters VA Medical Center, BRONX, NY USA

## Abstract

**Background:**

Neuropsychiatric symptoms (NPS) are core features of Alzheimer’s disease (AD) and have significant impact on patients, caregivers, and families. Worse NPS scores are associated with worse function and higher need for care. It is unclear if individual NPS symptoms may differentially affect functional decline and may be more effectively targeted to improve patient outcomes.

**Method:**

Data are drawn from participants enrolled in the National Alzheimer’s Coordinating Center Uniform Data Set (9/2005‐11/2022). Participants were diagnosed with Mild Cognitive Impairment or AD at baseline, had a primary etiologic diagnosis of AD, and had at least one annual follow‐up visit (average follow‐up = 4 years). Participants’ function was measured using the Functional Assessment Questionnaire (FAQ). NPS were reported by ADC study clinicians using the “Clinician Judgment of Symptoms” (Form B9) evaluating whether each of the following symptoms manifested as a meaningful change in behavior (yes = 1, no = 0): apathy‐withdrawal, depressed mood, visual or auditory hallucinations, delusions, disinhibition, irritability, agitation, and anxiety. Dementia severity was measured using the CDR. Multivariable analyses of the effects of each NPS on decline in function were performed using linear mixed models. Covariates included baseline age, gender, race/ethnicity, education, referral source, number of visits, comorbidities, APOE genotype, and number of medications.

**Result:**

Baseline sample characteristics (N = 9,358): mean age = 74±9, 47% male, 78% non‐Hispanic white, 11% black, 8% Hispanic, education = 15±4; 52% CDR = 0.5, 35% CDR = 1, and 13% CDR≥2; FAQ = 12±9. Baseline presence of NPS: apathy (34%), depressed mood (33%), anxiety (31%), irritability (28%), agitation (13%), disinhibition (10%), delusions (9%), and hallucinations (5%). Apathy was the most persistent of NPS with 37% of all participants had clinician endorsed apathy in ≥50% all of their visits, followed by depressed mood (28%), irritability (27%), anxiety (21%), agitation (14%), disinhibition (11%), delusions (9%), and hallucinations (5%). Functional decline was faster in those with persistent (≥50% of all visits) apathy, agitation, and delusions.

**Conclusion:**

Different behavioral symptoms have differential effects on functional decline, a major driver of caregiver stress and institutionalization. Better understanding of these differential effects on function is crucial in designing trials for treatment in AD. Assessing for these behavioral symptoms should be a standard component of cognitive evaluations.

